# Cystotomy and Cystectomy

**Published:** 1890-05-03

**Authors:** 


### CYSTOTOMY AND CYSTECTOMY.

This operation, in one or other form which recent progress in abdominal surgery has rendered safe enough, is now recognised as the only certain means of getting rid of the extreme distress occasioned by the presence of gall stones. Dr. Credé, of Dresden, reports the results of seven cases in which he has lately performed cystotomy or cystectomy. In two cystotomies he followed the usual procedure, once without the formation of a permanent fistula, but in the other a small sinus remained for two years, not, however, discharging bile. Two were what he calls "ideal" cystotomies, in which the bladder was closed with catgut sutures and left in situ, while in three cystectomy, or the removal of the gall bladder, was necessary. In all the wound healed completely and rapidly, without any complications, the middle of the incision being left open and closely covered with iodoform gauze.

In one of the cystectomies a large concretion was found, after the bladder had been removed, in the hepatic duct close to the commencement of the cystic. In consequence of the adhesions formed between the ducts and the surface of the liver it was equally impossible to get it out through the cystic duct or into the ductus communis, from which he would have removed it by an incision which he would have closed with continuous suture; he therefore pressed it through the wall of the hepatic duct adjacent to the liver, with which adhesion would certainly be effected, the aperture being meanwhile closed by contact with that organ.

His conclusions are that in severe cases it is better to remove the gall bladder altogether; but when the cystic duct is pervious and the bladder free from disease and serviceable, the "ideal" operation, the mortality from which will doubtless soon become much less than it is at present, is decidedly to be preferred, though in aged and weak patients the older operation with establishment of a fistula for some time is probably the safer course.

